# Supplementary figures and images for: Extensive Decoupling of Metabolic Genes in Cancer
Source: PLoS Comput Biol. 2015 May 11;11(5):e1004176. doi: 10.1371/journal.pcbi.1004176 (PMC4427321; doi:10.1371/journal.pcbi.1004176)

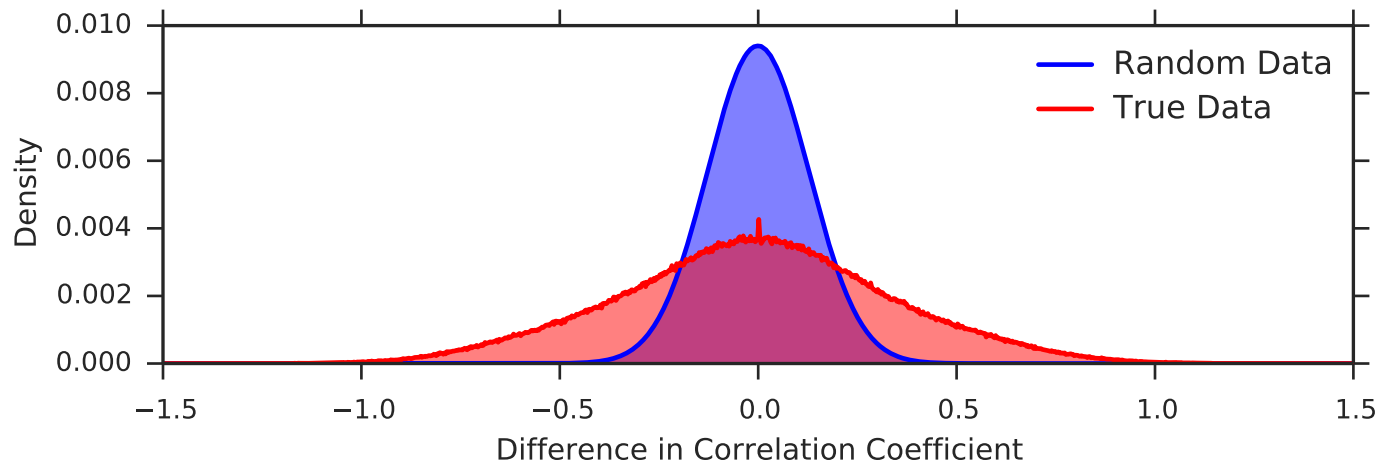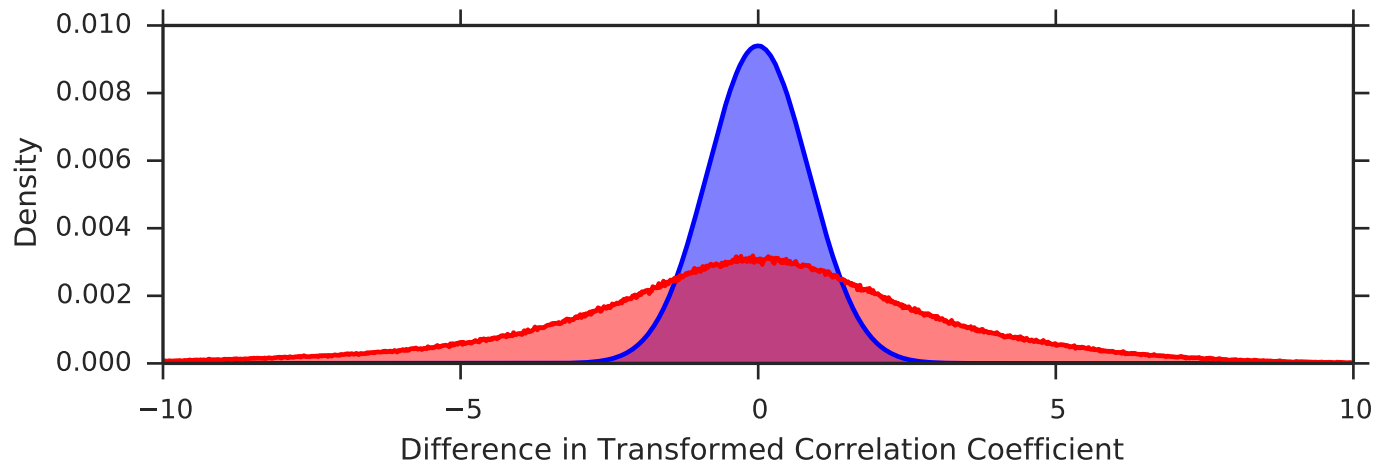

Supplement: S1 Fig — The labels (i.e. tumor or normal) of all RNA-Seq samples were permuted, and the difference in correlation coefficient calculated. This process was repeated 10000 times to generate a distribution. Differences in correlation coefficients tend to be larger in the true data, suggesting that differential co-expression is being observed. (PDF) [file pcbi.1004176.s002.pdf]

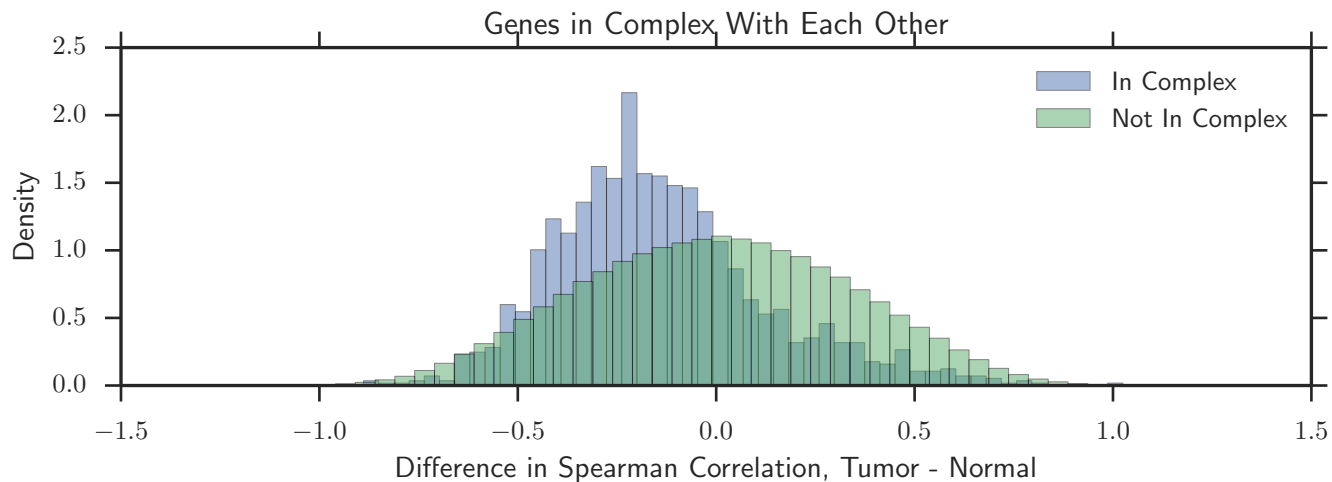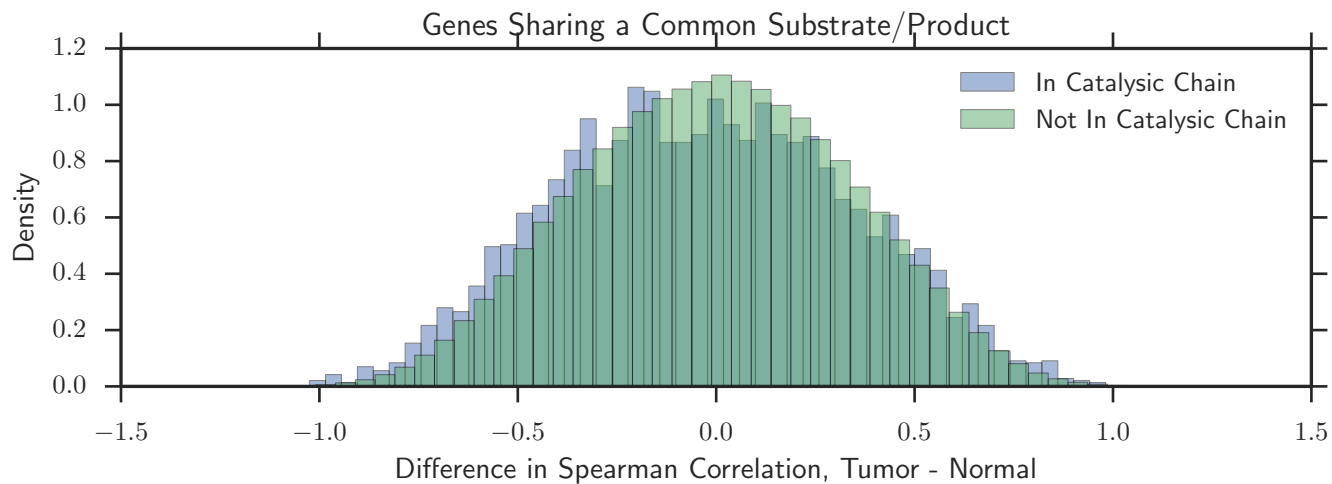

Supplement: S2 Fig — In top panel, a comparison is made between gene pairs whose products are in complex with each other, and gene pairs whose products are not in complex with each other. In the bottom panel, the unit of interaction is the “catalysis-precedes” binary interaction in Pathway Commons. A gene pair participates in this interaction if the gene products share a common substrate or product. Note that from the top panel, gene pairs whose products are members of a common complex show loss of co-expression in tumor samples. (PDF) [file pcbi.1004176.s003.pdf]

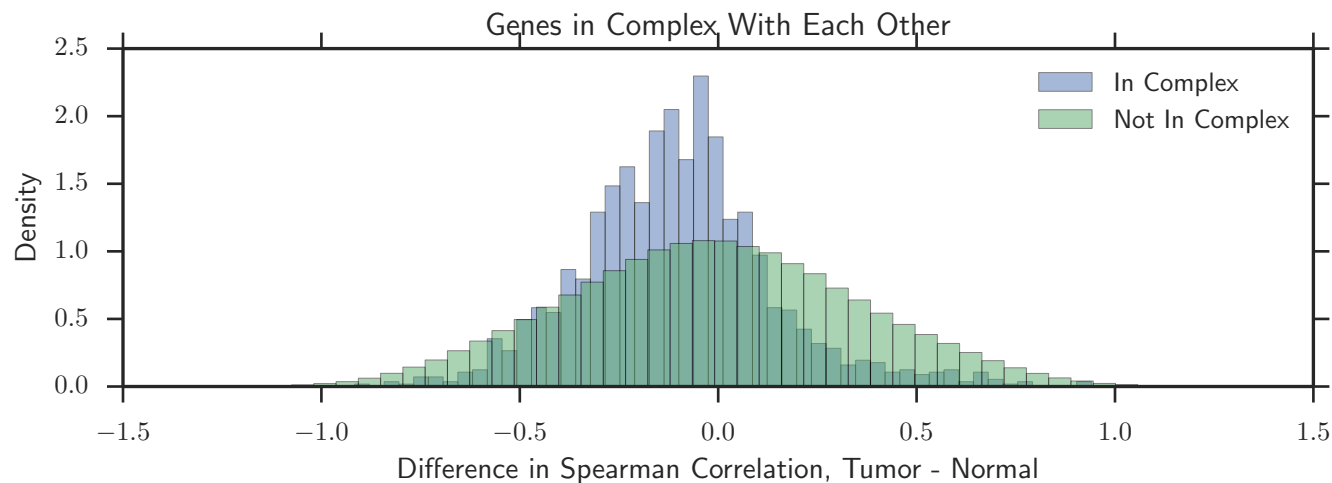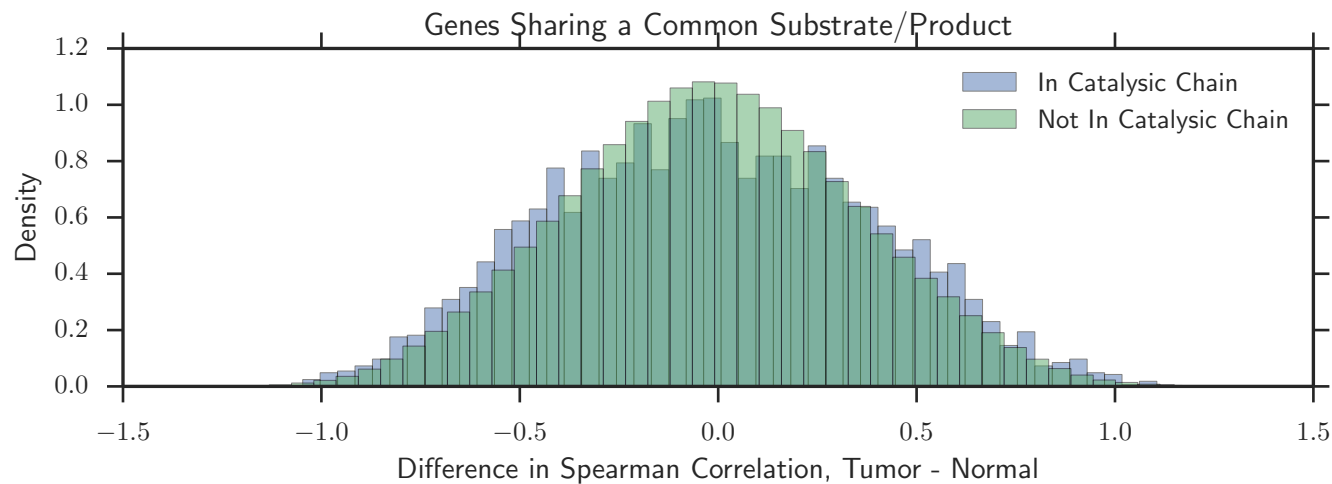

Supplement: S3 Fig — In top panel, a comparison is made between gene pairs whose products are in complex with each other, and gene pairs whose products are not in complex with each other. In the bottom panel, the unit of interaction is the “catalysis-precedes” binary interaction in Pathway Commons. A gene pair participates in this interaction if the gene products share a common substrate or product. Note that from the top panel, gene pairs whose products are members of a common complex show loss of co-expression in tumor samples. (PDF) [file pcbi.1004176.s004.pdf]

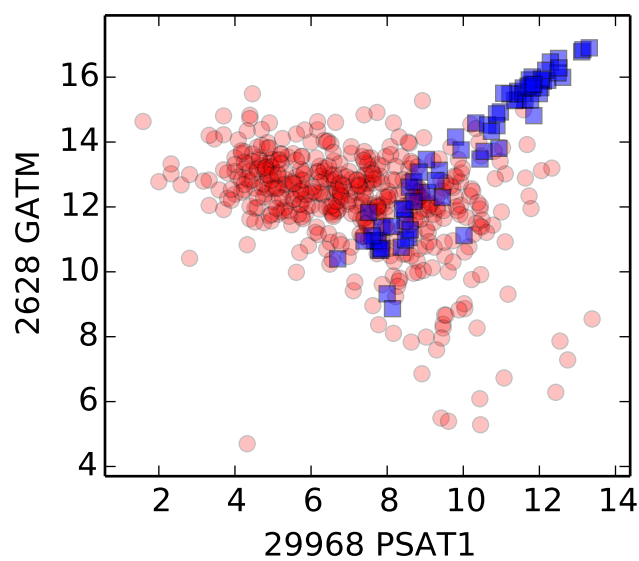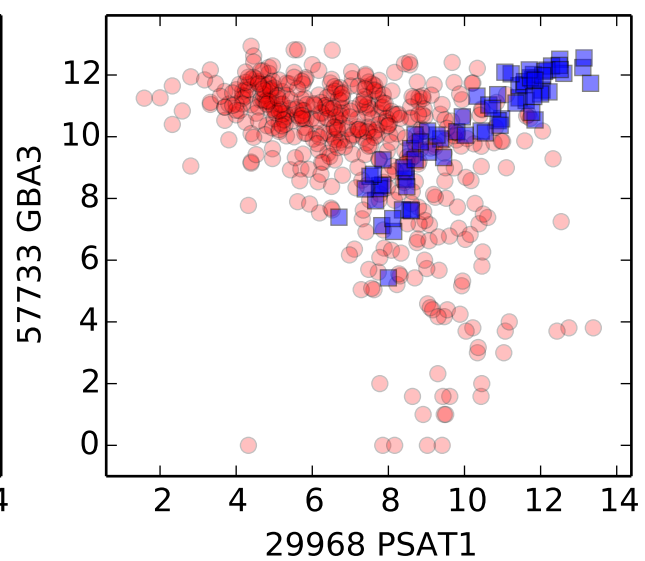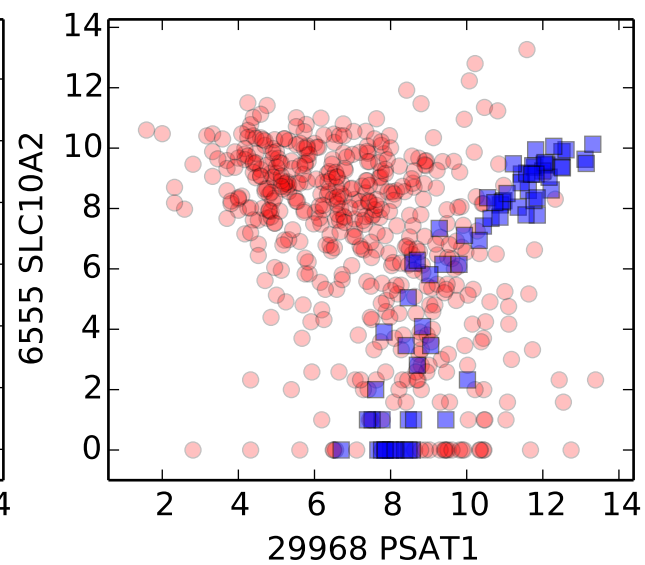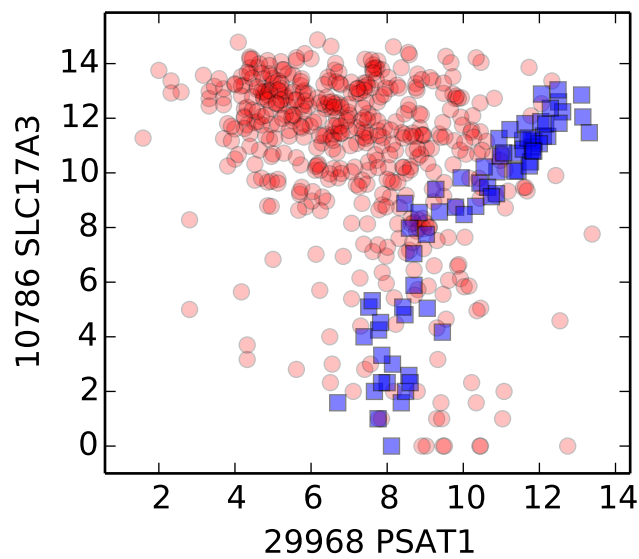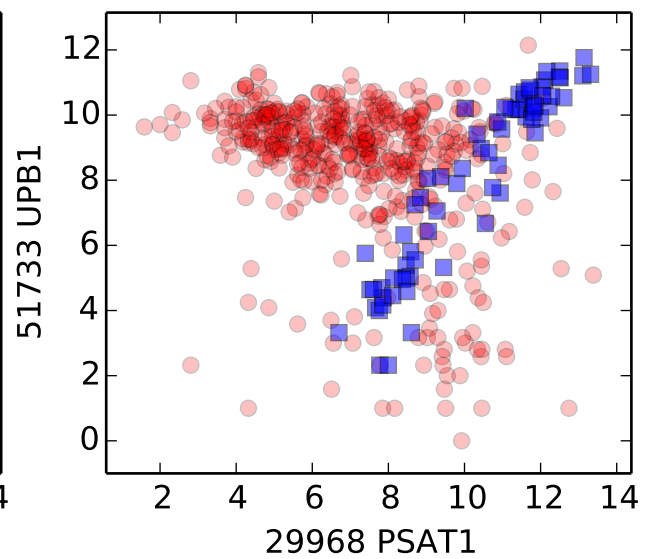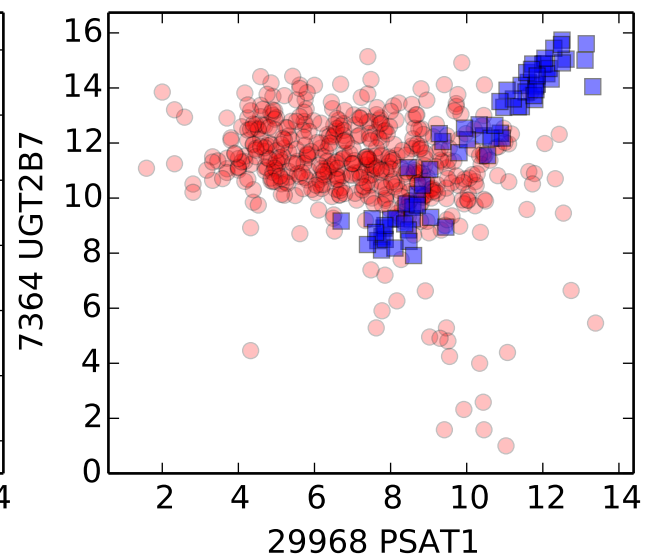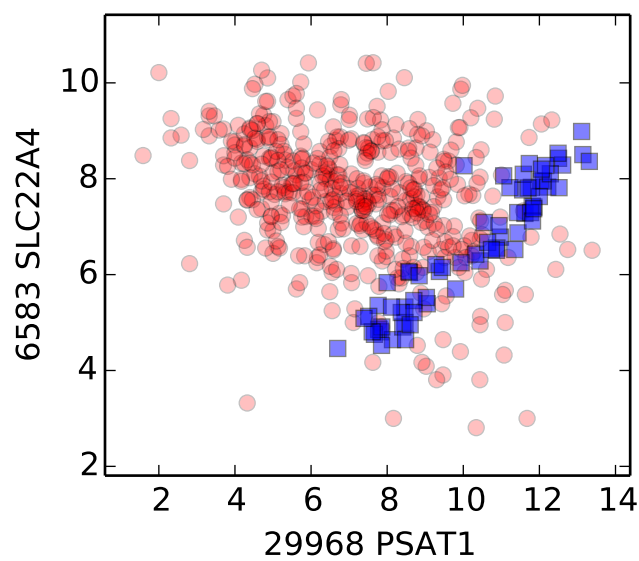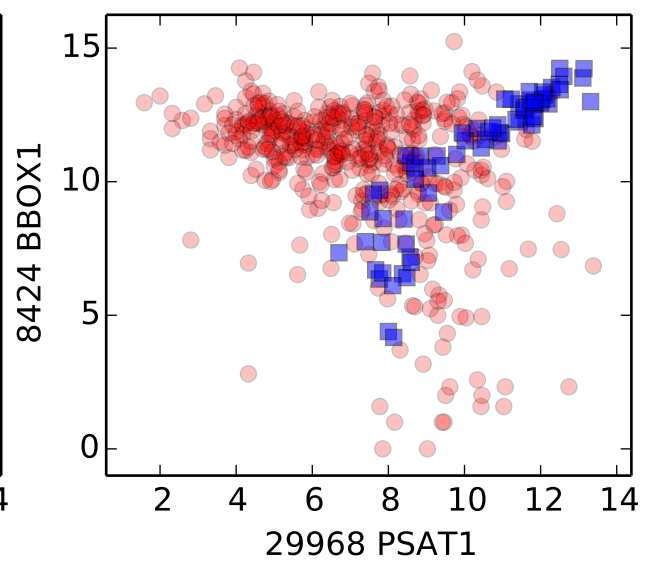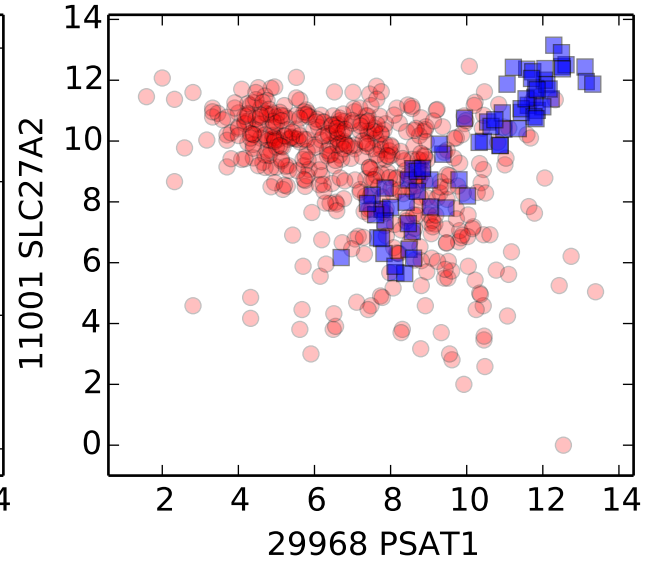

Supplement: S4 Fig — Blue dots correspond to normal tissue samples and red dots correspond to tumor samples. (PDF) [file pcbi.1004176.s005.pdf]

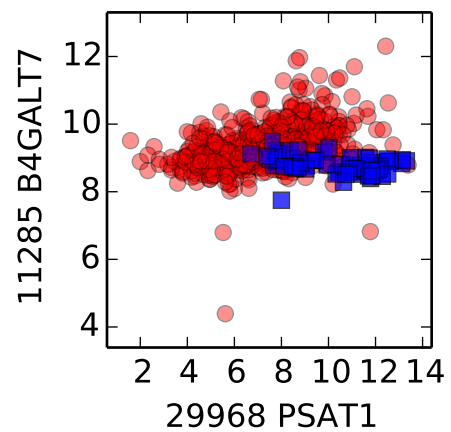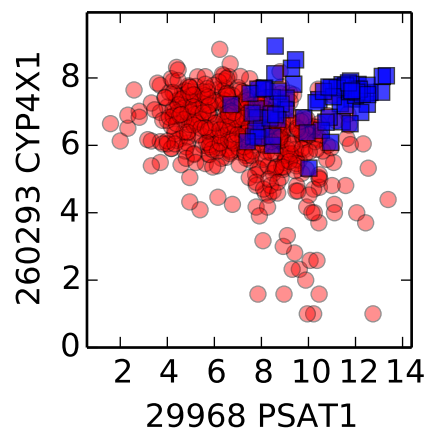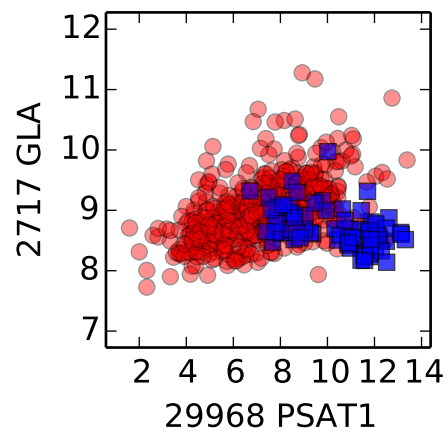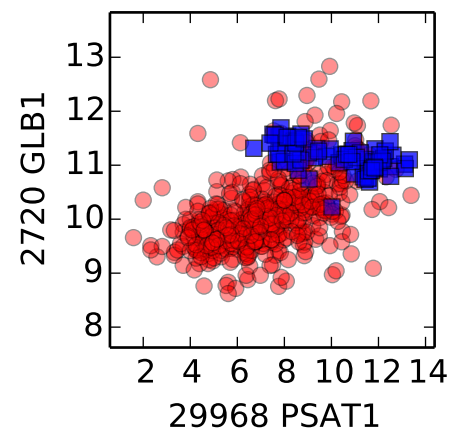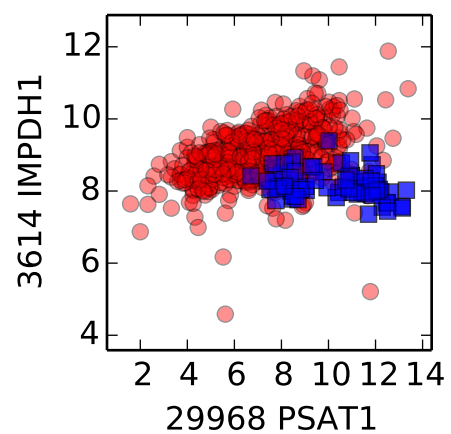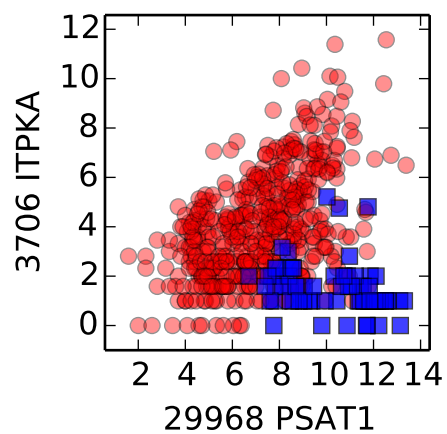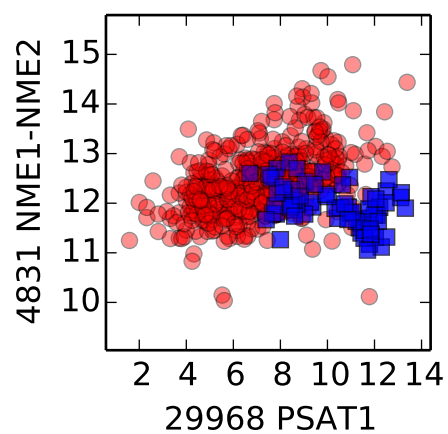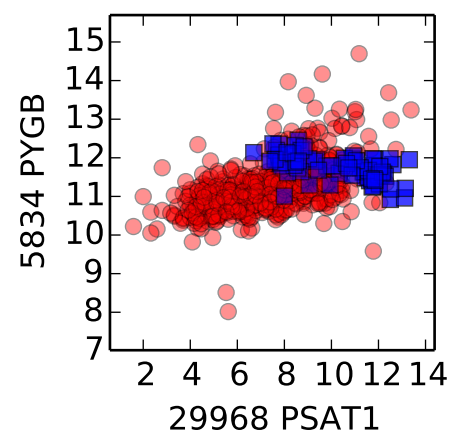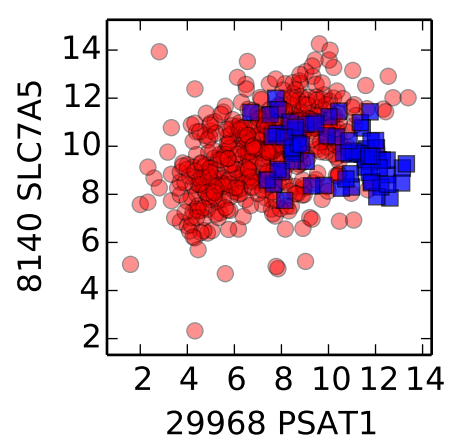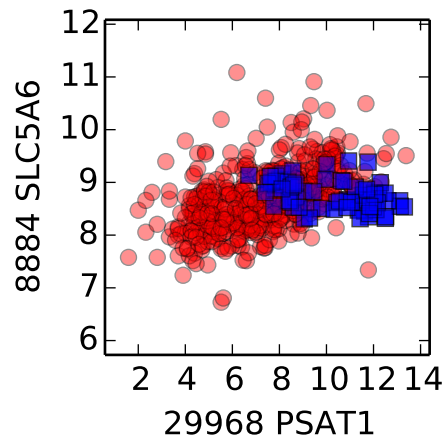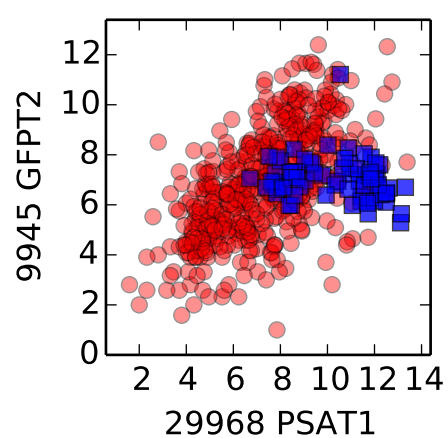

Supplement: S5 Fig — Blue dots correspond to normal tissue samples and red dots correspond to tumor samples. (PDF) [file pcbi.1004176.s006.pdf]

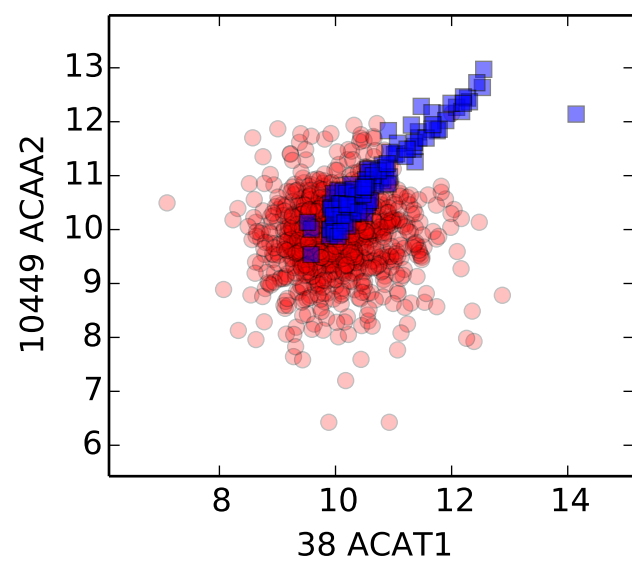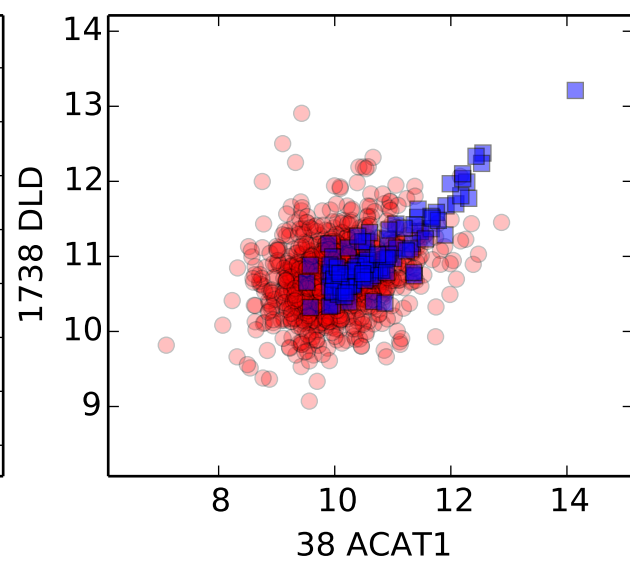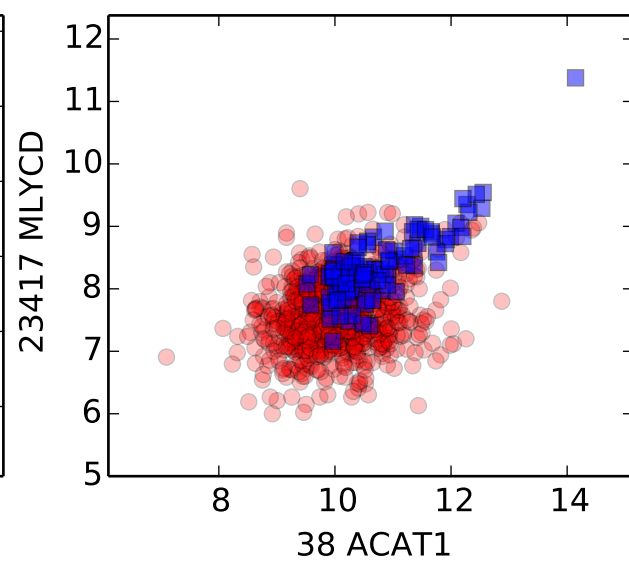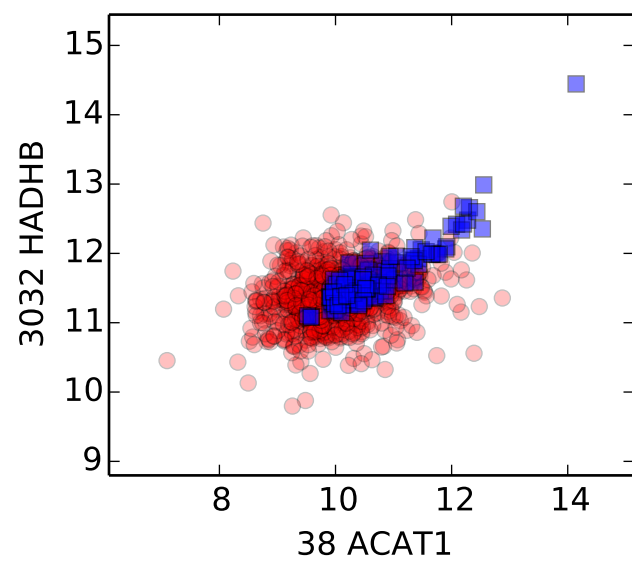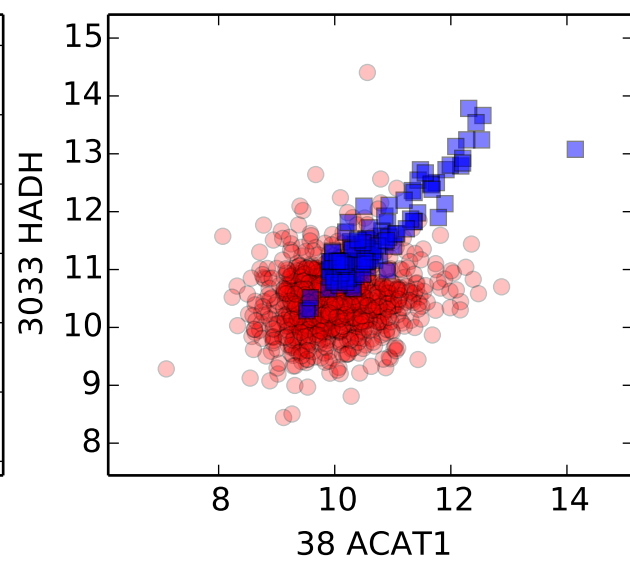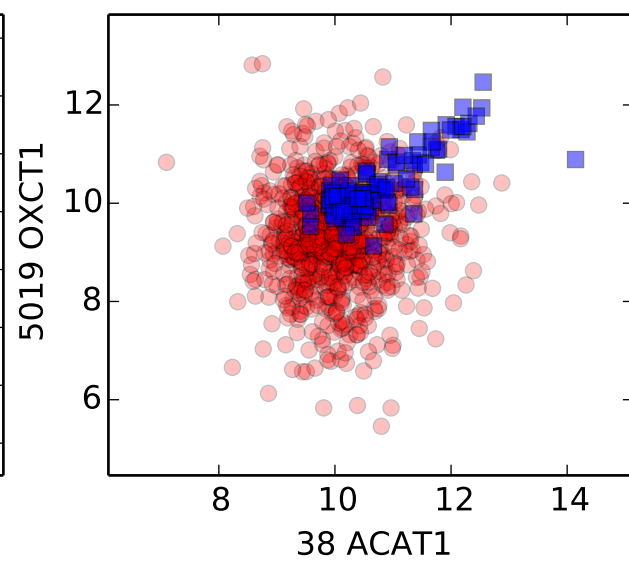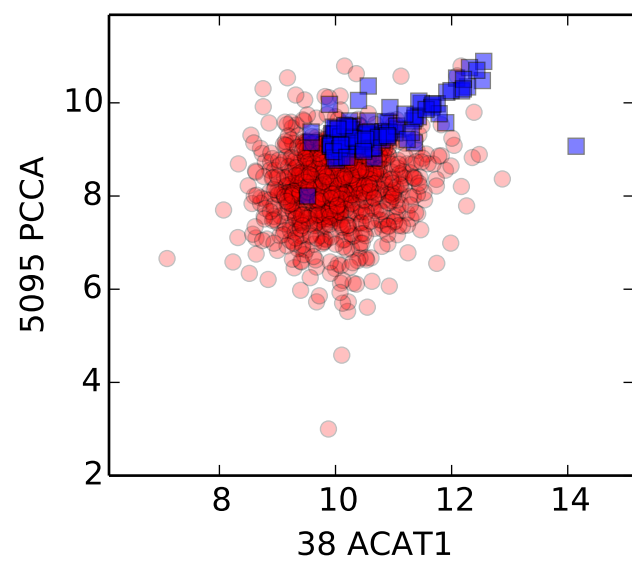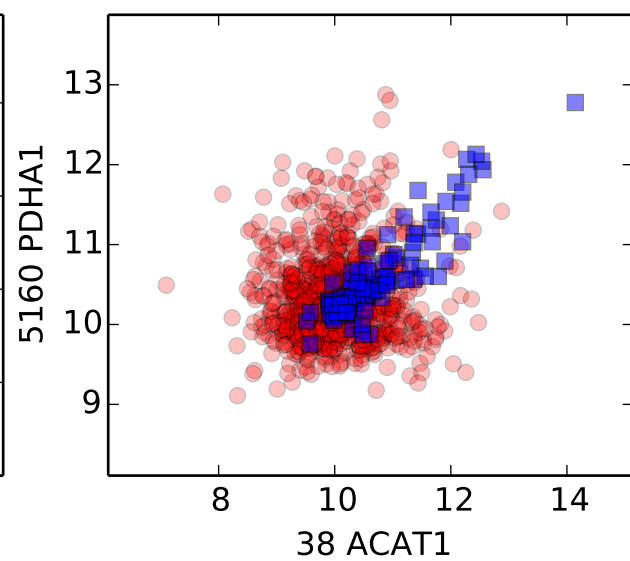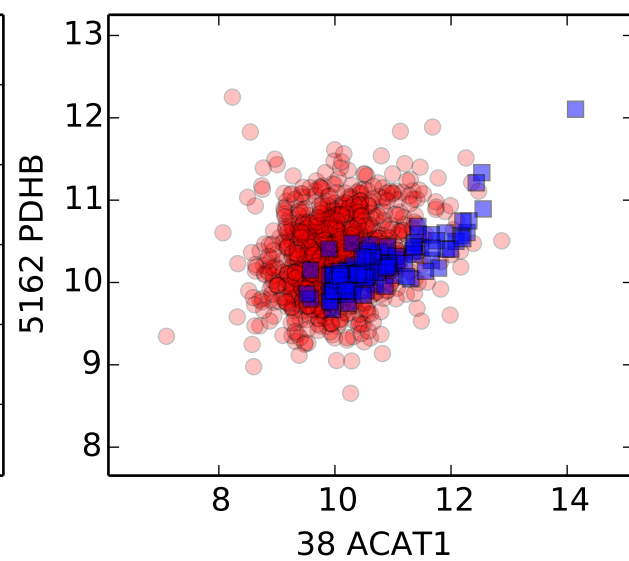

Supplement: S6 Fig — Blue dots correspond to normal tissue samples and red dots correspond to tumor samples. (PDF) [file pcbi.1004176.s007.pdf]

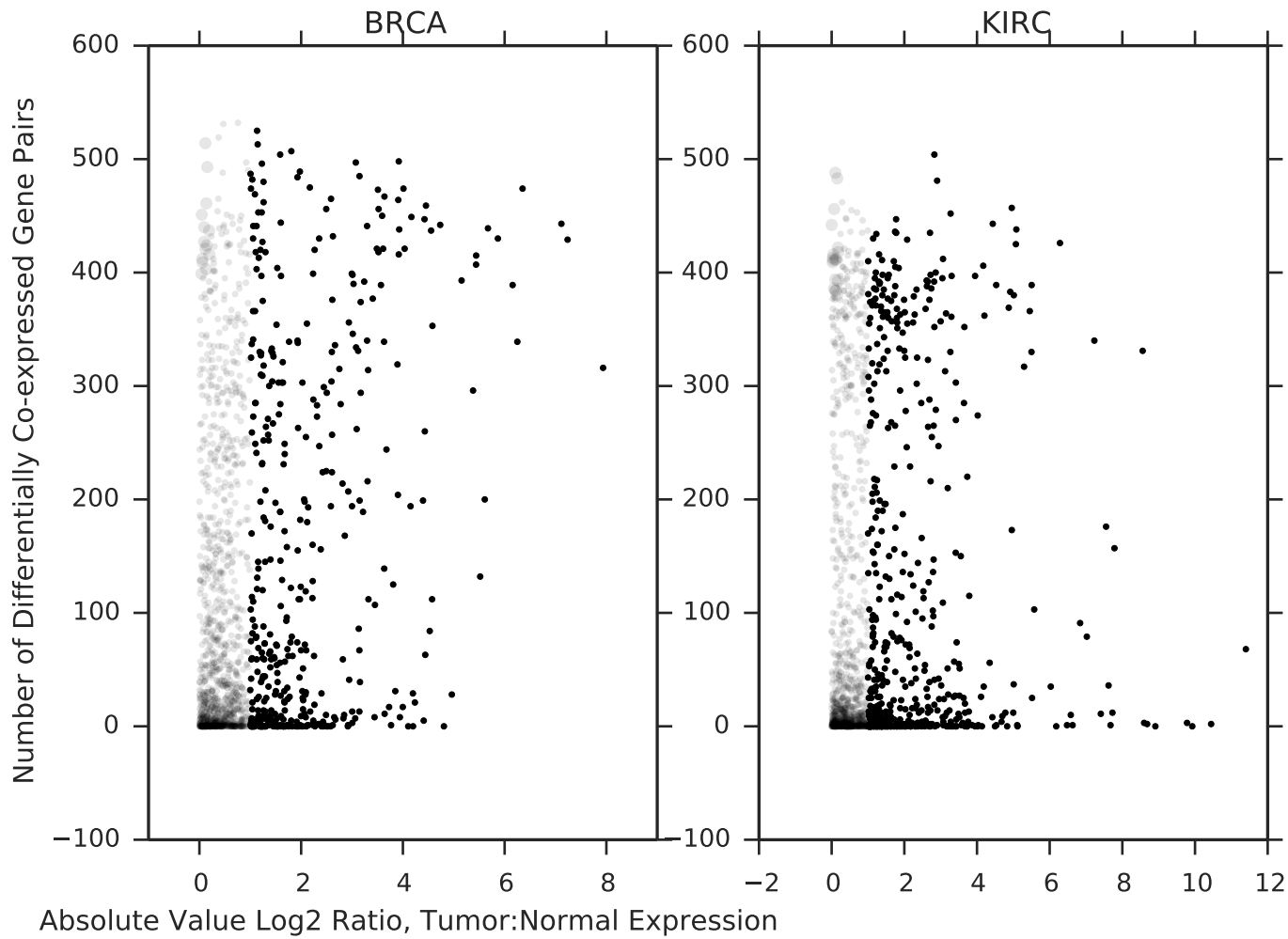

Supplement: S7 Fig — X-axis corresponds to the absolute value of the log2 ratio of expression between tumor and normal tissues. (PDF) [file pcbi.1004176.s008.pdf]

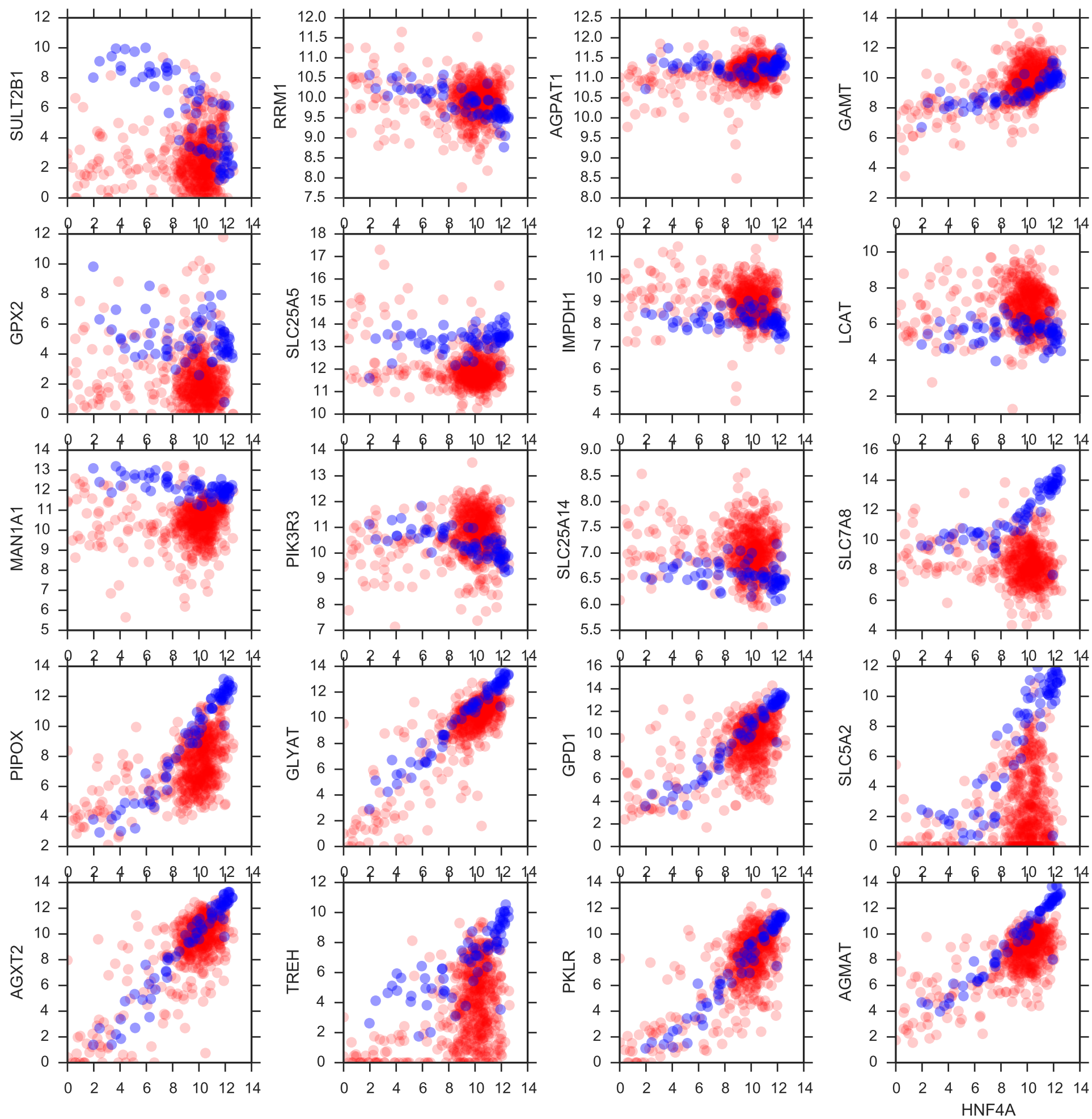

Supplement: S8 Fig — Blue dots correspond to normal tissue samples and red dots correspond to tumor samples. (PDF) [file pcbi.1004176.s009.pdf]
